# Interspecific differences in the responses of root phosphatase activities and morphology to nitrogen and phosphorus fertilization in Bornean tropical rain forests

**DOI:** 10.1002/ece3.8669

**Published:** 2022-03-07

**Authors:** Yu Hirano, Kanehiro Kitayama, Nobuo Imai

**Affiliations:** ^1^ 13126 Department of Forest Science Tokyo University of Agriculture Setagaya Japan; ^2^ 12918 Graduate School of Agriculture Kyoto University Kyoto Japan

**Keywords:** fertilization experiment, mycorrhizal type, phosphatase activity, resource partitioning, root morphology, successional status

## Abstract

Soil organic phosphorus (P) compounds can be the main P source for plants in P‐limited tropical rainforests. Phosphorus occurs in diverse chemical forms, including monoester P, diester P, and phytate, which require enzymatic hydrolysis by phosphatase into inorganic P before assimilation by plants. The interactions between plant interspecific differences in organic P acquisition strategies via phosphatase activities with root morphological traits would lead to P resource partitioning, but they have not been rigorously evaluated. We measured the activities of three classes of phosphatases (phosphomonoesterase, PME; phosphodiesterase, PDE; and phytase, PhT), specific root length (SRL), root diameter, and root tissue density in mature tree species with different mycorrhizal associations (ectomycorrhizal [ECM] or arbuscular mycorrhizal [AM]) and different successional status (climax or pioneer species) in Sabah, Malaysia. We studied nitrogen (N)‐ and P‐fertilized plots to evaluate the acquisition strategies for organic P under P‐limited conditions 7 years after fertilization was initiated. P fertilization reduced the PME activity in all studied species and reduced PhT and PDE activities more in climax species than in the two pioneer species, irrespective of the mycorrhizal type. PDE activity increased in some climax species after N fertilization, suggesting that these species allocate excess N to the synthesis of PDE. Moreover, PME and PhT activities, but not PDE activity, correlated positively with SRL. We suggest that climax species tend to be more strongly dependent on recalcitrant organic P (i.e., phytate and/or diester P) than pioneer species, regardless of the mycorrhizal type. We also suggest that trees in which root PME or PhT activity is enhanced can increase their SRL to acquire P efficiently. Resource partitioning of soil organic P would occur among species through differences in their phosphatase activities, which plays potentially ecologically important role in reducing the competition among coexisting tree species in lowland tropical rainforests.

## Graphical abstract

We investigated the response of root morphological traits and activities of three classes of root phosphatase to nitrogen and phosphorus (P) fertilization for seven dominant tree species in Bornean tropical rain forest to verify the interspecific difference in organic P acquisition strategies. Climax species were more dependent on more recalcitrant organic P than pioneer species, regardless of mycorrhizal types (ectomycorrhizal or arbuscular mycorrhizal). Our results suggest that resource partitioning for soil organic P occur among tree species through differences in their phosphatase activities in P‐limited lowland tropical rainforests.

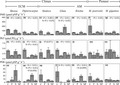

## 1 INTRODUCTION

Typical niche theory assumes that species diversity advances through trade‐offs that lead to the partitioning of limiting resources among species. This means that different species use unique acquisition strategies to acquire a resource in limited supply (Tilman, 2004). It is well known that species may be specialized in their capacity to acquire different chemical forms of soil nitrogen (N) at N‐limited sites, such as in temperate and arctic ecosystems (Kahmen et al., 2006; McKane et al., 2002; Miller & Bowman, 2002; Zhou et al., 2021). A similar phenomenon may occur with soil phosphorus (P), insofar as P resources exist in various organic forms that require different types of enzymatic hydrolysis before they can be exploited by plants (Liu et al., 2018; Turner, 2008).

Phosphorus, which is originally supplied from bedrock, is widely thought to limit the biological processes of plants in lowland tropical rainforests because the weathering and leaching processes here are intense (Vitousek, 1984). Under such conditions, the main supply of P available to plants may shift from the inorganic P pool to the hydrolyzed organic P pool (Cabugao et al., 2021; Imai et al., 2010; Kitayama, 2013). There are various forms of organic P, which must be hydrolyzed to inorganic P by different classes of phosphatase enzymes before the P can be taken up by plants as a mixture of monoester P (including labile monoester P and phytate) and diester P, which is the most abundant form in tropical soils (Turner & Engelbrecht, 2011). Labile monoester P (e.g., glucose phosphate, mononucleotides) and phytate (salts of myo‐inositol hexakisphosphate) are hydrolyzed by the enzyme phosphomonoesterase (PME) and a special class of PME, called “phytases” (PhTs), respectively. Diester P (e.g., that in DNA and RNA) is hydrolyzed by both PME and phosphodiesterase (PDE) to inorganic P, which is more costly to acquire by plants than labile monoester P (Turner & Haygarth, 2005). The acquisition of phytate probably incurs the highest metabolic cost among the organic P compounds because phytate must be solubilized before its enzymatic hydrolysis, as it is robustly stabilized on mineral soil surfaces (Turner, 2008). Plant species may increase their expression of different classes of phosphatases (PME, PDE, and PhT) to depend on these different forms of organic P under P‐limited conditions. This may lead to resource partitioning for soil organic P among species (Ahmad‐Ramli et al., 2013; Turner, 2008; Yokoyama et al., 2017).

Acquisition strategies for soil organic P may differ among species of different successional status and mycorrhizal types (Yokoyama et al., 2017). It is possible that climax species, which dominate primary forests, have a stronger ability than pioneer species to use organic P other than labile monoester P, reducing the competition for organic P (Huang et al., 2013). This is because belowground competition for organic P by plants will be greater in the later stages of forest succession, with the accumulation of soil nutrients in wood biomass during this process and reduced cycling rates of limiting nutrients (Huang et al., 2013; Tang et al., 2011). Furthermore, trees that form associations with ectomycorrhizal (ECM) fungi may depend more on soil organic P than arbuscular mycorrhizal (AM‐associated) tree species (Phillips & Fahey, 2006; Rosling et al., 2016). This is because ECM fungi have evolved from saprotrophs and are known to exploit not only inorganic P but also organic P via the exudation of phosphatase enzymes (Phillips & Fahey, 2006; Smith & Read, 2008), whereas AM fungi are thought to mainly increase the acquisition of inorganic P only (Dodd et al., 1987; Joner et al., 2000; Tarafdar & Marschner, 1994). Turner (2008) specifically predicted that mycorrhizae are likely to play a central role in the partitioning of P by plants, and Liu et al. (2018) suggested that ECM‐associated trees depend more on organic P sources, including recalcitrant phytate, than AM‐associated trees. Phenomena such as P resource partitioning among plant species have been reported in temperate peatlands (Ahmad‐Ramli et al., 2013), grasslands (Ceulemans et al., 2017; Phoenix et al., 2020), and tropical forests (Liu et al., 2018; Steidinger et al., 2015). However, most studies that have investigated P resource partitioning among species have used experiments with seedlings grown for very short periods. They have rarely examined phosphatase activities directly (e.g., Liu et al., 2018) or have focused on limited classes of phosphatases, and few have examined the effect of differences in successional status.

In addition to the physiological traits of roots, such as their phosphatase activities, many studies have investigated the adaptive mechanisms of root morphological traits. In general, plant species that have fine roots with a high specific root length (SRL; root length per root weight) have a strong capacity to acquire nutrients (Aerts & Chapin, 2000; Ostonen et al., 2007). Therefore, SRL or the specific root surface area (root surface area per root weight, an index that correlates positively with SRL) may increase as the availability of soil P decreases along a natural soil P gradient (Cabugao et al., 2021; Powers et al., 2005; Ushio et al., 2015). It is also suggested that PME activity correlates significantly positively with SRL or the specific root surface area, allowing the plant to take up soil P efficiently, and that both SRL and PME activity increase tropical tree species under low‐P conditions (Lugli et al., 2020; Ushio et al., 2015). Although two fertilization studies have investigated the adaptive responses of root morphology to P fertilization at the plot scale (Lugli et al., 2021; Wurzburger & Wright, 2015), no study has yet examined the interspecific differences in morphological root adaptations in response to nutrient fertilization or the relationships between morphological traits and phosphatase activities other than PME (e.g., PhT and PDE).

In a study conducted by Yokoyama et al. (2017), which was a precursor to the present study, they measured three classes of fine‐root phosphatase (PME, PhT, and PDE) using bulk soil/root samples from mixed species in the same factorial NP‐fertilized plots as we used in the present study. They found that P fertilization reduced the PME and PhT activities (but not PDE activity) at the stand level and suggested that most tree species do not depend greatly on diester P as a P source at the stand level in Bornean tropical rainforests. However, they did not examine interspecific differences in the adaptation of root phosphatase activities to nutrient limitation. Therefore, the partitioning of soil organic P between species in lowland, species‐rich, tropical rainforests remains unclear.

We measured the activities of three classes of phosphatase (PME, PDE, and PhT) and the root morphologies (root diameter, root tissue density, and SRL) of seven mature tree species with different mycorrhizal associations (ECM or AM) and successional status (climax or pioneer) using factorial NP‐fertilized plots in Sabah, Malaysia. We tested the following four hypotheses: (1) P fertilization reduces the PDE and/or PhT activities in climax species but not in pioneer species, as climax species have a stronger capacity to use diester P and/or phytate to avoid competition for soil organic P in late successional stages; (2) P fertilization reduces the PhT activities of ECM‐associated species but not AM‐associated species because ECM‐associated species depend more on recalcitrant organic P (phytate) than AM species (Liu et al., 2018); (3) N fertilization enhances the phosphatase activities in ECM species because these species may allocate excess N to the synthesis of extracellular phosphatases (proteins that have high N contents) to acquire P, which is reflected in the high phosphatase activity in ECM‐associated roots (Marklein & Houlton, 2012; Phillips & Fahey, 2006; Treseder & Vitousek, 2001); and (4) P fertilization reduces SRL in all target species, resulting in the simultaneous reduction of phosphatase activity and SRL after P fertilization, as tropical trees invest in both increased phosphatase activity and SRL under P‐limited conditions (e.g., Lugli et al., 2020).

## 2 MATERIALS AND METHODS

### 2.1 Study sites

The study sites were located in Deramakot Forest Reserve and Tangkulap Forest Reserve in Sabah, Malaysian Borneo (5°14–30′N, 117°11–36′E) (Imai et al., 2009, 2010, 2012; Yokoyama et al., 2017). The climate is humid equatorial. The mean annual temperature is 25.2°C and the annual precipitation is 3098 mm (Ong et al., 2013). The soils are characterized as acrisols (Sabah Forestry Department, 2005). The natural forests in the two reserves are composed largely of overlogged mixed lowland dipterocarp forests (Ong et al., 2013). Tangkulap Forest Reserve, in particular, has been highly damaged by intensive logging (Imai et al., 2012; Ong et al., 2013).

Twelve 0.12 ha (30 m × 40 m) plots were established in primary forest in Deramakot Forest Reserve and another 12 plots in secondary forest in Tangkulap Forest Reserve by N. Imai, unpublished data. The primary forests are dominated by climax species, such as those of the family Dipterocarpaceae, whereas the secondary forests are dominated by pioneer species of the genus *Macaranga* (Euphorbiaceae). An experiment comparing four treatments with factorial NP fertilization (control, +N, +P, +NP, *n* = 3 each) for each forest type was initiated in December 2011. A total of 24 plots (2 forest types ×4 treatments ×3 replicates) were used for the experiment. Urea as the source of N and triple superphosphate (TSP) as the source of P were scattered by hand at rates of 100 kg N ha^−1^ and 50 kg P ha^−1^, respectively. Care was taken to fertilize the entire area of each plot homogeneously. Each 0.12 ha plot was divided into 12 subplots of 0.01 ha (10 m × 10 m), and a fixed amount of fertilizer was applied evenly to each subplot. Fertilization was continued annually at the same rate from 2011. Detailed information on the soil chemistry and the fluxes of CO_2_, CH_4_, and N_2_O in the fertilized plots have been described by Yokoyama et al. (2017), Mori et al. (2017), and Mori et al. (2018). The effects of the NP fertilization strategy on the ecosystem structure and functions will be reported elsewhere (Imai et al., in preparation).

We selected seven evergreen broad‐leaved species, representing a broad range of the taxa of the commonest woody species in our study sites. Five of the seven species (*Shorea multiflora*, *Dipterocarpus acutangulus*, *Sindora irpicina*, *Gluta wallichii*, and *Knema latericia*) are defined as climax species because these species dominate primary forests. *Shorea multiflora* and *D*. *acutangulus* are canopy‐dominant dipterocarp species. *Sindora irpicina*, *G*. *wallichii*, and *K*. *latericia* are sub‐canopy non‐dipterocarp species. Two of the seven species (*Macaranga pearsonii* and *M*. *gigantea*) are pioneer species, which dominate secondary forests. Symbiotic fungi are AM for most tree species in lowland tropical rainforests (Alexander, 1989). In contrast, dipterocarp species form symbiotic associations with ECM fungi (Alexander and Lee, 2005). *Sindora* is a genus of the family Leguminosae, but it is a non‐nodulating (non‐N_2_‐fixing) genus (Afkhami et al., 2018). Therefore, *S*. *multiflora* and *D*. *acutangulus* are ECM‐associated species, whereas *G*. *wallichii*, *K*. *latericia*, *S*. *irpicina*, *M*. *pearsonii*, and *M*. *gigantea* are AM‐associated species.

### 2.2 Fine‐root sampling

We selected a total of 86 trees of the 7 target species within the experimental plots with diameters at breast height (dbh) ranging from 7.4 to 81.8 cm (Table S1). Fine‐root samples were collected from August to November 2019 after annual nutrient applications had been applied nine times (5 months after the last application). It is difficult to sample the fine roots of large adult trees in tropical rainforests because there are various root systems of many species in the soil (Aoki et al., 2012). Therefore, we sampled the fine roots of the target species from many saplings (0.3–1.5 m tall), prepared the root specimens (Figure S1), and learned the colors, textures, sizes, wound exudates, and branching patterns of the roots and conducted several trials to sample the fine roots from large trees to understand the locations at which the fine roots attach to the larger roots. Ultimately, we could confidently identify and sample the fine roots from large trees of the target species. We excavated the fine‐root systems (<2 mm diameter) of each tree using a manual shovel and then a trowel and skewer. The fine‐root systems were isolated carefully from the soil and organic matter to ensure that they were not damaged. The collected samples were immediately taken back to the laboratory and stored in a refrigerator at 4°C until enzymatic analysis. All enzyme assays were conducted within 3 days of root sampling.

### 2.3 Phosphatase analysis

The root PME and PDE activities were measured using the method of Antibus et al. (1992), Treseder and Vitousek (2001), and McLachlan (1980), with some modifications. The measurement of root PhT activities was based on the modified method of George et al. (2008) and Yokoyama et al. (2017). We could verify the interspecific differences in phosphatase activities between ECM‐ and AM‐associated species because this assay evaluates the activities of surface‐bound and extracellular acid phosphatases associated directly with the plant roots and the fungal mantle (in ECM‐associated plants, but not in AM‐associated species) (Antibus et al., 1992; Steidinger et al., 2015).

To measure the root PME activities, the soil particles and leaf litter attached to the collected root samples were washed off briefly with tap water. Four to five root subsamples (40–100 mg each, approximately first‐ to fourth‐order roots), which contained root caps, were removed from the primary root sample and transferred into a 2 ml microtube (designated S1, S2, S3, [S4], and P for each subsample, *n* = 3 or 4). We also prepared a 2 ml microtube without any root subsample as the control (designated C). Subsamples S1, S2, S3, P, and C received 1.75 ml of acetate buffer (pH 5.0) containing NaN_3_. Subsamples S1, S2, S3, and C then received 0.25 ml of 40 mM *p*‐nitrophenyl phosphate (pNPP) in acetate buffer as the substrate for the enzyme reactions, and subsample P received 0.25 ml of 5 mM *p*‐nitrophenol (pNP). These subsamples were incubated in the dark at 25°C for 30 min, and then 1 ml of the reaction solution was transferred to a 6 ml glass vial containing 0.5 ml of 0.5 M NaOH and 4.0 ml of pure water to stop the reaction and develop the color of the *p*‐nitrophenol released as the product of the enzymatic reaction. The assay mixture was then shaken and the absorbance measured with a spectrophotometer at a wavelength of 410 nm (Tabatabai & Bremner, 1969). The absorbance was compared with a standard curve of pNP and converted to the amount of pNP generated during the incubation of the subsample.

The same procedure was used to measure the root PDE activity, but with a substrate solution of 40 mM bis‐nitrophenyl phosphate (bis‐NPP) in pure water.

To measure the root PhT activity, six root subsamples (40–100 mg each), which contained root caps, were removed from the primary root sample, and each was transferred into a 2 ml microtube (designated S1, S2, S3, C, P, and N, *n* = 3). We also prepared a 2 ml microtube with no root subsample (designated B_1_ or B_2_ for each subsample). All subsamples received 0.75 ml of acetate buffer (pH 5.0) containing NaN_3_. Subsamples S1, S2, S3, and B_1_ then received 0.25 ml of 8 mM phytic acid sodium salt in acetate buffer as the enzyme reaction substrate. Subsample C, N, and B_2_ received 0.25 ml of acetate buffer. Subsample P received 0.25 ml of 10 mM KH_2_PO_4_. The subsamples were incubated in the dark at 25°C for 14 h, and the enzymatic reaction was terminated by the addition of 0.06 ml of 100% trichloroacetic acid (TCA). Subsamples S1, S2, S3, P, N, and B_1_ then received 0.94 ml of acetate buffer, whereas subsamples C and B_2_ received 0.69 ml of acetate buffer and 0.25 ml of 8 mM phytic acid sodium salt in acetate buffer. Each assay mixture was then shaken, and 1 ml of the reaction solution was used to determine the concentration of orthophosphate using the molybdenum blue method. The absorbance at 712 nm was measured on a spectrophotometer (MP‐1200, Erma Inc.).

The activities of root PME and PDE were determined as:

$$\mathrm{Activities}=\;\frac{S‐C}{T\times P/\mathrm{Pb}}\left(\mu \mathrm{mol}\;\mathrm{pNP}\;g^{‐1}\;h^{‐1}\right)$$

The activities of root PhT were determined as:

$$\mathrm{Activities}=\;\frac{S‐C‐B_{1}+B_{2}}{T\;\times \;(P‐N)/\mathrm{Pb}}\left(\mu \mathrm{mol}\;{\mathrm{PO}}_{4}\;g^{‐1}\;h^{‐1}\right)$$

*S*, Mean amount of pNP or PO_4_ in the solutions of subsamples S1, S2, and S3 after incubation per g dry weight of the subsample (μmol g^−1^); *C*, Amount of pNP or PO_4_ in the solution of subsample C after incubation per g dry weight of subsample (μmol g^−1^); *B*_1,_ Amount of PO_4_ in the solution of subsample B_1_ after incubation per g dry weight of subsample (μmol g^−1^); *B*_2_, Amount of PO_4_ in the solution of subsample B_2_ after incubation per g dry weight of subsample (μmol g^−1^); *P*, Amount of pNP or PO_4_ in subsample P after incubation per g dry weight of subsample (µmol g^−1^); *N*, Amount of PO_4_ in subsample N after incubation per g dry weight of subsample (µmol g^−1^); *T*, Incubation time (h); Pb, Amount of pNP or PO_4_ added to subsample P before incubation per g dry weight of subsample P (µmol g^−1^).

### 2.4 Root morphology

After the PME, PDE, and PhT activities were measured, the root subsamples in which they were measured were washed briefly with tap water and placed in a laboratory dish. The laboratory dish was placed on a double‐lamp bed scanner (GT‐X970, Epson), and a digital image of the root subsample was taken. The image was used to calculate the mean root diameter (mm), total root length (cm), total surface area (cm^2^), and total volume (cm^3^) using ImageJ (https://rsbweb.nih.gov/ij/) and the macro program IJ_Rhizo (https://www.quantitative‐plant.org/software/IJ_Rhizo), an image analysis system designed specifically for root measurement (Pierret et al., 2013). The mean root diameter, total root length, and total root volume were estimated by assuming that the roots were cylindrical in structure. The root samples were then dried in an oven at 65°C for 48 h, and the dry root weight was measured. SRL (m g^−1^) and the root tissue density (g cm^−3^) were calculated from the total length, root volume, and dry mass of the samples. The values for 13–15 replicate root subsamples for each individual tree were averaged. In total, the root morphology of 1130 root subsamples (13–15 root subsamples ×2–5 individuals ×4 treatments ×7 species) was analyzed. The average values were taken as the value for an individual plant and used for downstream statistical analyses.

### 2.5 Statistical analysis

The free statistical environment R was used (R Core Team 2021) for all analyses. We used linear mixed models (LMMs) to assess the significant effects of treatment and species on the classes of root phosphatase activities and the root morphology using the lmer function in the lmerTest package (Kuznetsova et al., 2017). We treated an individual tree as a random effect and N and P fertilizer treatments and species as fixed effects in the LMMs. The model equation was as follows: root phosphatase activity (PME, PDE, or PhT) or root morphology (root diameter, root tissue density, or SRL) ~ treatment (control or P fertilization or N fertilization or NP fertilization) + species +interaction between treatment and species +random effect (individual tree). We also used LMMs for each species to assess the significant effects of P fertilization and N fertilization on the classes of root phosphatase activities detected and root morphology. The model equation used was as follows: root phosphatase activity (PME, PDE, or PhT) or root morphology (root diameter, root tissue density, or SRL) ~ P fertilization (P fertilization or non‐P fertilization) + N fertilization (N fertilization or non‐N fertilization) + interaction between P fertilization and N fertilization +random effect (individual tree). Because we could not obtain adequate residual plots, PME, root diameter, root tissue density, and SRL were log‐transformed before analysis. The interspecific differences in phosphatase activities and SRL in the control plots were also analyzed using LMMs. We treated individual trees as a random effect and species as a fixed effect. Post hoc tests were performed using the function glht in the multcomp package (Hothorn et al., 2008), using Tukey contrast for multiple comparisons to assess interspecific differences in the control plots.

A series of three analyses of covariance (ANCOVA) models was used for the individual‐level analyses, using PME, PhT, or PDE activity as the dependent variable, fertilization treatment as a factor, and SRL as a covariate.

Using the mean values for 3–5 mature trees per plant species grown in P‐fertilized or control plots, we calculated the percentage differences in root phosphatase activities (PME, PhT, and PDE) and SRL. The percentage difference was calculated as the difference between the P‐fertilized and control plots for each trait value divided by the average of the trait value in the control plots, multiplied by 100. The relationship between the percentage difference in the root phosphatase activity (PME, PhT, or PDE) and the percentage difference in SRL was evaluated using a linear regression analysis.

## 3 RESULTS

### 3.1 Root phosphatase activity

**FIGURE 1:**
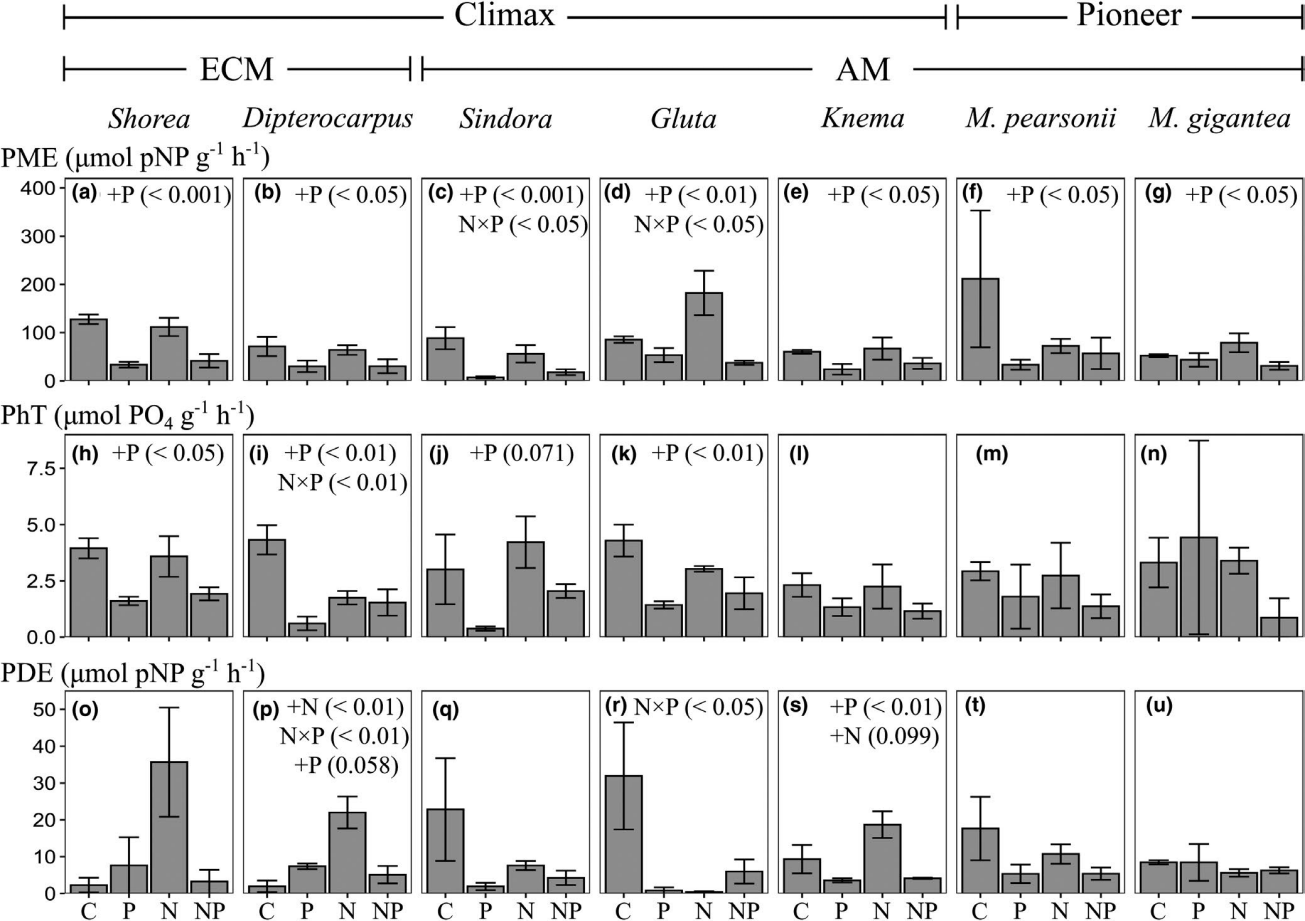
PME activity (a–g), PhT activity (h–n), and PDE activity (o–u) in seven dominant tree species in the control, N, P, and N and P fertilization plots (C, N, P, and NP, respectively) in lowland tropical rainforest in Borneo. Bars represent geometric means ± SE. ECM or AM indicate ectomycorrhizal or arbuscular mycorrhizal type, respectively. Letters (+N, +P, or N × P) indicate significant overall N and P effects or an N × P interaction effect, followed by linear mixed model *p* values

The root phosphatase activities did not differ significantly among the seven species studied, regardless of the phosphatase class (Figure S2a–c). Phosphorus fertilization significantly reduced the PME activity in all seven species (Figure 1a–g). Similarly, fertilization tended to alter the PhT activity, and the effect did not differ among species (Table S2). However, among the target species, P fertilization reduced the PhT activity more markedly in the four climax species (excluding *Knema*) than in the two pioneer species studied even though the interaction between fertilization and species is not significant in the statistical model (Figure 1h–n, Table S2). Moreover, P fertilization reduced the PDE activity significantly only in *Knema* (Figure 1s), and the response of PDE activity to fertilization differed significantly among species (interaction between treatment and species for PDE: *p* < .05; Table S2).

Nitrogen fertilization had no significant effect on the PME or PhT activity in any of the seven species (Figure 1a–n). However, it increased the PDE activities in *Dipterocarpus* (an ECM‐associated tree species) and *Knema* (an AM‐associated tree species) significantly (Figure 1p,s).

### 3.2 Root morphology

All morphological traits (SRL, root diameter, and root tissue density) and the responses of all morphological traits to fertilization differed significantly among the species (Table S2).

**FIGURE 2:**
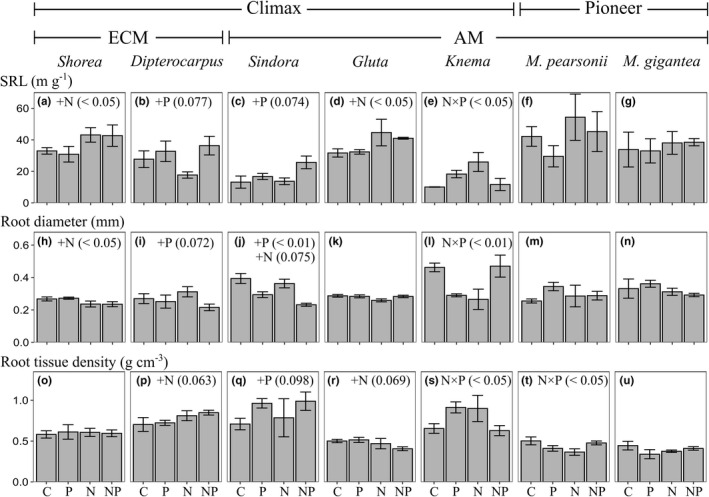
Specific root length (SRL) (a–g), root diameter (h–n), and root tissue density (o–u) in seven dominant tree species in the control, N, P, and N and P fertilization plots (C, N, P, and NP, respectively) in lowland tropical rainforest in Borneo. Bars represent geometric means ± SE. ECM or AM indicate ectomycorrhizal or arbuscular mycorrhizal type, respectively. Letters (+N, +P or N × P) indicate significant overall N and P effects or an N × P interaction effect, followed by linear mixed model *p* values

In the control plots, there were significant differences in SRL among the species according to post hoc Tukey tests, and *Knema* showed the lowest SRL values (Figure S2d). Contrary to our hypothesis, N or P fertilization increased SRL significantly or marginally in the climax species other than *Knema* (*Shorea*, *Dipterocarpus*, *Sindora*, and *Gluta*; Figure 2a–e). In contrast, no significant effects of N or P fertilization (separately or in combination) were detected in the two pioneer species (Figure 2f,g).

In general, the increase in SRL is caused by a reduction in root diameter and/or root tissue density (Ostonen et al., 2007). The root diameter of *Gluta* was not affected by fertilization, but the root tissue density decreased after N fertilization (Figure 2k,r). The root diameter decreased significantly or marginally in climax species other than *Gluta* (*Shorea*, *Dipterocarpus*, and *Sindora*), but the root tissue density was unchanged or tended to increase after N and/or P fertilization (Figure 2h–j,l,o–q,s).

### 3.3 Relationship between root phosphatase activity and SRL

**FIGURE 3:**
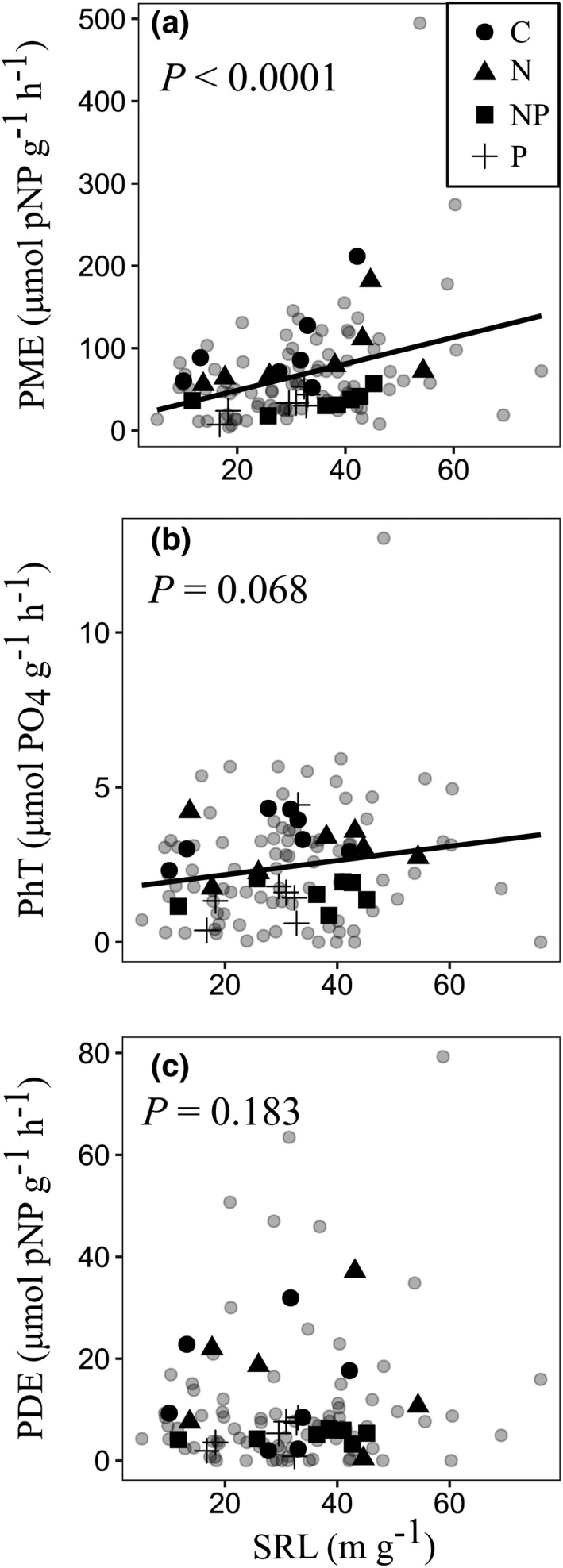
Relationships between root phosphatase activity (PME, PhT, or PDE) and specific root length (SRL) at the levels of individual trees and species (a, b, c). Gray circles indicate values at the level of individuals. Solid line indicates the regression line estimated using linear model analysis. *p* Values are results for SRL in the ANCOVA model

We examined the relationships between phosphatase activity (PME, PhT, and PDE) and SRL at the level of individual trees (*n* = 86) to verify the general trends across the target species. PME activity and SRL showed a significant positive correlation (SRL: *p* < .0001; treatment: *p* < .0001; Figure 3a). PhT activity and SRL showed a marginally positive correlation (SRL: *p* < .068; treatment: *p* < .001, Figure 3b). However, PDE activity and SRL showed no significant correlation (SRL: *p* = .183; treatment: *p* < .01; Figure 3c).

**FIGURE 4:**
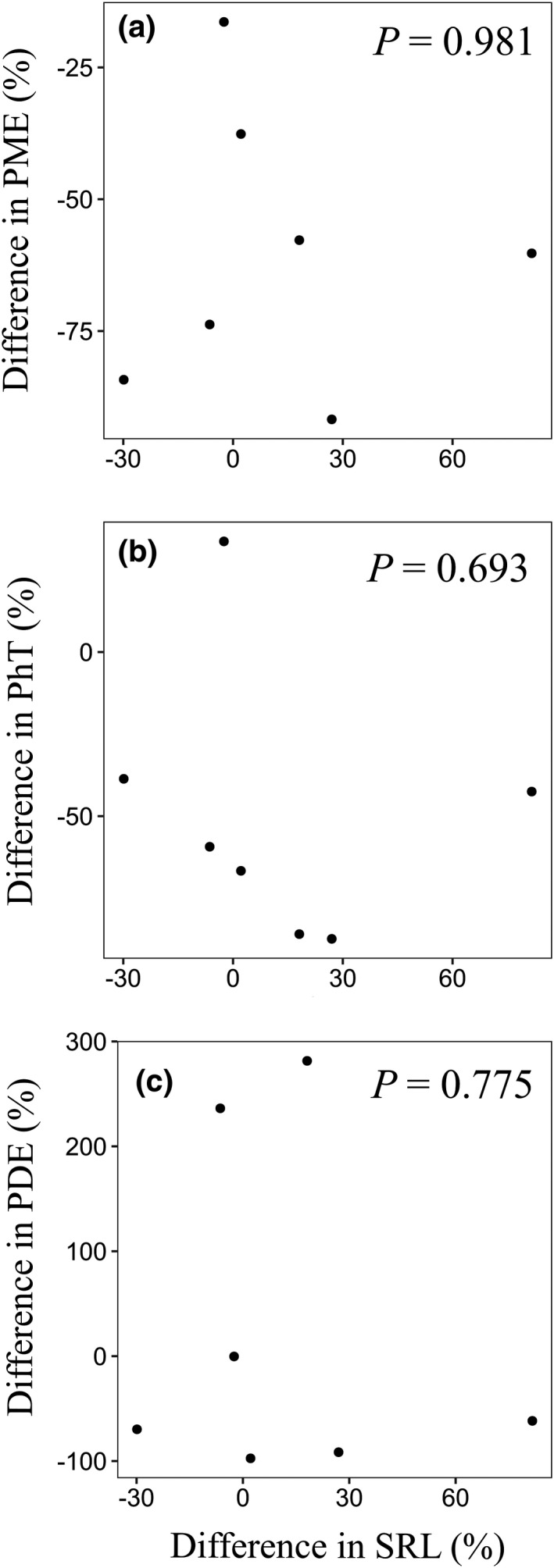
Relationships between the percentage difference in root phosphatase activity (PME, PhT, or PDE) and the percentage difference in specific root length (SRL) in response to P fertilization at the species level (a, b, c). It is not significantly correlated across all figures

We also examined the relationships between the difference in root phosphatase activity (PME, PhT, and PDE) and the difference in SRL in response to P fertilization at the level of the tree species (using trait values in the P fertilization and control plots). However, we found no significant relationship between the differences in root phosphatase activity and SRL in response to P fertilization, regardless of the phosphatase class (PME, PhT, or PDE; Figure 4a–c).

## 4 DISCUSSION

### 4.1 Responses of phosphatase activities to P fertilization

Only a few similar studies have reported the root phosphatase activities of tropical trees. Our PME values for all seven species in the control plots (50–210 µmol pNP g^−1^ h^−1^) were close to those of tropical tree species on Mount Kinabalu, Borneo, which ranged from 90 to 180 pNP g^−1^ h^−1^ (Kitayama, 2013; Ushio et al., 2015). Most tropical tree species are essentially dependent on labile organic P, but their dependence on more recalcitrant organic P differs according to their successional status. Fertilization with P significantly reduced the PME activity in all seven species (Figure 1a–g; Table S2), indicating that most tropical tree species depend on labile monoester P, as shown in previous P fertilization experiments (Treseder & Vitousek, 2001; Yokoyama et al., 2017; Zheng et al., 2015). In contrast, a reduction in PhT activity following P fertilization was observed in four of five climax species (*Shorea*, *Dipterocarpus*, *Sindora*, and *Gluta*), but not in the two pioneer species (Figure 1h–n), although the change in PhT activity with fertilization did not differ significantly among species (Table S2). P fertilization also reduced the PDE activity in *Knema*, one of the five climax species (Figure 1s), but not in the two pioneer species (Figure 1m,n,t,u). Based on these results for PhT and PDE activities, climax species may depend more strongly on recalcitrant organic P than do pioneer species. Huang et al. (2013) reported higher PME activities in species that dominated in the later stages of forest succession rather than in those that dominated the early stages and suggested that competition among plants for P is greater in later successional stages. Thus, in such P‐limited conditions, climax species may have a potential to avoid competition for P among species through depending on recalcitrant organic P other than labile monoester P (i.e., phytate and/or diester P), the acquisition of which incurs more metabolically high cost.

We detected no difference in the PhT activities of climax and pioneer species in the control plots (Figure S2b), and the climax species tended to depend more on phytate than did the pioneer species (Figure 1h–n). In contrast, Yokoyama et al. (2017) reported that the fine roots in secondary forests had higher PhT activity than those in primary forests, suggesting that the pioneer trees in secondary forests depend more on phytate than the climax trees in primary forests. These differences between the studies probably arise from the fact that even within pioneer species, different types of acquisition strategies for organic P could be used to acquire phytate. Typical pioneer species (including the two *Macaranga* species studied; Aoyagi et al., 2013) do not depend on phytate, whereas some pioneer species may have a strong capacity to acquire phytate via PhT activity. Future research should examine the variation in the P acquisition strategies of pioneer species in a larger number of species.

Fertilization with P reduced the PhT activities not only in ECM‐associated species (*Shorea* and *Dipterocarpus*) but also in AM‐associated species (*Sindora* and *Gluta*) (Figure 1h–k). This indicates that ECM and some AM species depend on recalcitrant phytate. Previous studies have shown inconsistent findings on the dependence of AM tree species on recalcitrant organic P. For example, in an experiment in which seedlings were fertilized using different chemical forms of P, Liu et al. (2018) showed that ECM tree species exploited more recalcitrant organic P (phytate) than AM tree species, indicating soil P partitioning between ECM and AM tree species. By contrast, Steidinger et al. (2015) reported that ECM and AM species exploit similar forms of organic P, and Moyersoen et al. (2001) suggested that AM tree species are actually distributed under conditions of high soil organic nutrients, as are ECM species. We have not directly compared the extracellular phosphatase activities of mycelia and could not evaluate the hyphal networks of AM fungi in this study. However, our study shows that AM species depend on recalcitrant organic P, like ECM species, which suggests that even AM species utilize organic P via phosphatases exudated directly by plant roots. Therefore, our results suggest that resource partitioning for soil organic P does not occur between ECM and AM trees in this tropical forest in Malaysian Borneo.

### 4.2 Responses of phosphatase activities to N fertilization

Nitrogen fertilization had no significant effect on the PME or PhT activity in any of the seven species examined (Figure 1a–n). This suggests that these species do not allocate excess N to the synthesis of PME or PhT. Because phosphatase proteins have relatively high N contents (8%–32%), an increase in phosphatase activity after N fertilization has been reported (Marklein & Houlton, 2012; Treseder & Vitousek, 2001; Zheng et al., 2015). However, some studies have shown that N fertilization does not increase phosphatase activity, even in tropical lowland forests (Lugli et al., 2021; Turner & Joseph Wright, 2014; Yokoyama et al., 2017). Specifically, Yokoyama et al. (2017) reported that N fertilization did not increase the root phosphatase activity of any of the three phosphatase classes (PME, PhT, and PDE) and concluded that these Bornean lowland forests are saturated with N. Our results also demonstrate that most tree species in our lowland tropical forests invest sufficient amounts of N in the synthesis of PME and/or PhT.

However, N fertilization increased PDE activity in *Dipterocarpus* (an ECM species) and *Knema* (an AM species; Figure 1p,s), suggesting that the two species allocate excess N to the synthesis of PDE to acquire soil P. In *Knema*, in particular, fertilization with P significantly reduced the PDE activity, but not PhT activity (Figure 1s), indicating that *Knema* depends more strongly on diester P than the other species examined. To avoid competition with coexisting species, *Dipterocarpus* and *Knema* may invest more N in the synthesis of PDE (but not in PME or PhT) to acquire diester P, upon which the other tree species do not depend. In tropical forests, where diverse species coexist, several tree species may acquire different P resources to avoid competition among species (Liu et al., 2018; Nasto et al., 2017; Steidinger et al., 2015; Turner, 2008).

### 4.3 Relationship between root morphology and root phosphatase activity

We predicted that P fertilization would reduce SRL in all the target species because plant species with high SRL may be better able to acquire nutrients (Aerts & Chapin, 2000; Ostonen et al., 2007). Previous studies have reported that the fine‐root length/biomass and the specific root surface area (an index that correlates positively with SRL) increase as the availability of soil P decreases along a natural soil P gradient (Powers et al., 2005; Ushio et al., 2015). Surprisingly, N or P fertilization increased SRL in the four climax species other than *Knema*, but not in the pioneer species (Figure 2a–g). Moreover, the root diameter decreased in three climax species (*Shorea*, *Dipterocarpus*, and *Sindora*) following N and/or P fertilization, and the root tissue density of *Gluta* decreased following N fertilization (Figure 2h–j,r). Similarly, Wurzburger and Wright (2015) also demonstrated that the fertilization of N, P, and K together in tropical forests increased SRL at the stand level but reduced the root tissue density. Roots with high SRL and low tissue density are short lived and suited for rapid resource acquisition, but they incur the high costs associated with root tissue construction and maintenance (Aerts & Chapin, 2000; Gill et al., 2002). Given the P and/or N limitation in the late successional stages of tropical forests (Huang et al., 2013; Tang et al., 2011), the alleviation of nutrient limitation may cause the roots of climax species (but not those of pioneer species) to shift to the expression of functional traits that produce short‐lived roots. Actually, it is known that the production of fine roots is faster in more fertile areas than in infertile areas (Doughty et al., 2014).

In this study, we found that PME and PhT activities correlated positively with SRL at the level of individual trees (Figure 3a,b). Recent studies have also reported a positive correlation between PME activity and SRL or the specific root surface area (Lugli et al., 2020; Ushio et al., 2015). By contrast, PDE activity and SRL did not correlate significantly (Figure 3c), probably because most species do not depend on diester P (Figure 1o–u). These results indicate that tropical trees that have roots with high SRL are associated with greater PME or PhT activities.

However, there was no relationship between the difference in phosphatase activity and the difference in SRL in response to P fertilization at the species level (Figure 4a–c). This is because, as mentioned earlier, SRL unexpectedly increased or remained unchanged following P fertilization (Figure 2a–g). This may be attributable to phylogenetic effects on these morphological traits. In a global meta‐analysis, it was reported that some root morphological traits, including SRL, are more strongly constrained by phylogenetic structuring than by environmental factors (Valverde‐Barrantes et al., 2017). Therefore, root morphology and phosphatase activity may not have changed simultaneously in response to P fertilization because phylogenetic and/or species‐specific constraints affect the acclimation responses of root morphological traits to soil nutrient concentrations.

*Knema* and *Dipterocarpus*, whose PDE activities increased following N fertilization, may have different P acquisition strategies from those of the other species. *Knema* in the control plots showed the highest root diameter and the lowest SRL (Figure S2d and Figure 2e,l), indicating that *Knema* has thicker roots than other species. Furthermore, the root morphological traits of *Knema* did not change after NP fertilization (Figure 2e,l,s). Therefore, *Knema* may invest N to acquire P by secreting phosphatases (PDE), rather than by extending its roots to scavenge soil P. Phosphorus fertilization reduced the PME and PhT activities of *Dipterocarpus* (Figure 1b,i). Therefore, *Dipterocarpus*, an ECM species, may have the capacity to exploit all major soil organic P by secreting PME, PhT, and PDE. This may be reflected in the higher phosphatase enzyme activities in ECM‐associated roots than in AM‐associated roots (Phillips & Fahey, 2006). These mechanisms would explain why *Dipterocarpus* is one of the canopy‐dominant species at this site. We suggest that these differences in organic P acquisition strategies may also reflect the resource partitioning of soil organic P among tree species, and that organic P strategies are species‐specific, but do not necessarily differ between ECM and AM species.

## 5 CONCLUSIONS

Our results indicate that climax species tend to be more strongly dependent upon recalcitrant organic P (phytate and/or diester‐P) than pioneer species in tropical rainforests. Moreover, some climax species acquire diester P by allocating excess N to the synthesis of PDE, independently of the mycorrhizal type. We also suggest that phosphatase activities correlate positively with SRL. We conclude that resource partitioning for soil organic P, as in the conceptual model of Turner (2008), occurs in tropical rainforests and reduces the competition among coexisting tree species. This process may play a potentially important ecological role in promoting the coexistence of tree species in Bornean lowland, species‐rich, tropical rainforests.

## CONFLICT OF INTEREST

The authors declare that they have no potential conflicts of interest.

## AUTHOR CONTRIBUTIONS

**Yu Hirano:** Conceptualization (equal); Data curation (lead); Formal analysis (lead); Funding acquisition (supporting); Investigation (lead); Methodology (equal). **Kanehiro Kitayama:** Conceptualization (equal); Data curation (supporting); Formal analysis (supporting); Funding acquisition (supporting); Investigation (supporting); Methodology (equal). **Nobuo Imai:** Conceptualization (lead); Data curation (supporting); Formal analysis (supporting); Funding acquisition (lead); Investigation (supporting); Methodology (supporting).

## Supporting information

Figure S1

Figure S2

Table S1

Table S2

## Data Availability

Data are available from the Dryad Digital Repository: https://doi.org/10.5061/dryad.905qfttm3 (Hirano et al., 2022).
