# Supplementary figures and images for: Interspecific differences in the responses of root phosphatase activities and morphology to nitrogen and phosphorus fertilization in Bornean tropical rain forests
Source: Ecol Evol. 2022 Mar 7;12(3):e8669. doi: 10.1002/ece3.8669 (PMC8901874; doi:10.1002/ece3.8669)

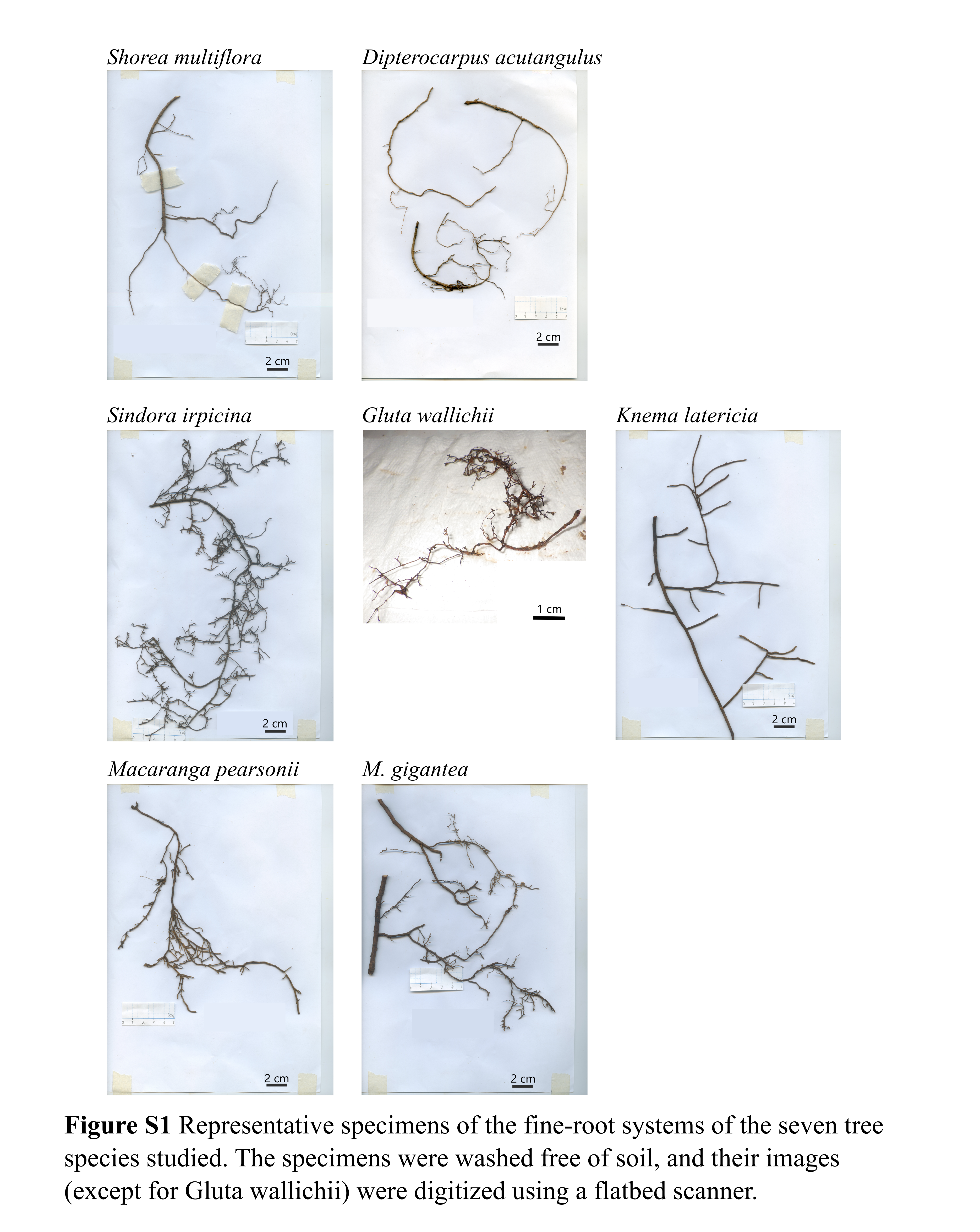

Supplement: Supplementary file 1 — Figure S1 [file ECE3-12-e8669-s003.tif]

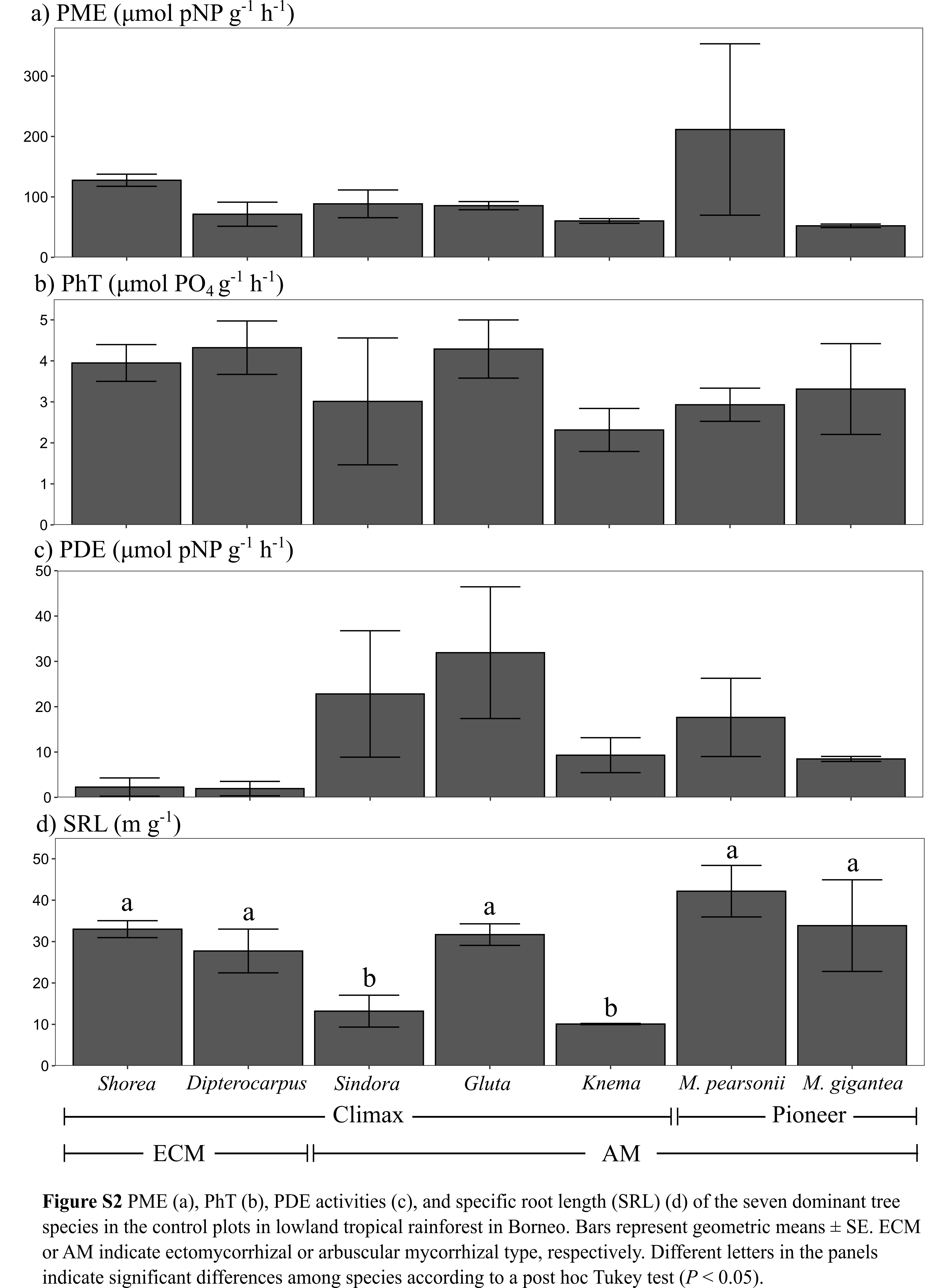

Supplement: Supplementary file 2 — Figure S2 [file ECE3-12-e8669-s002.tif]

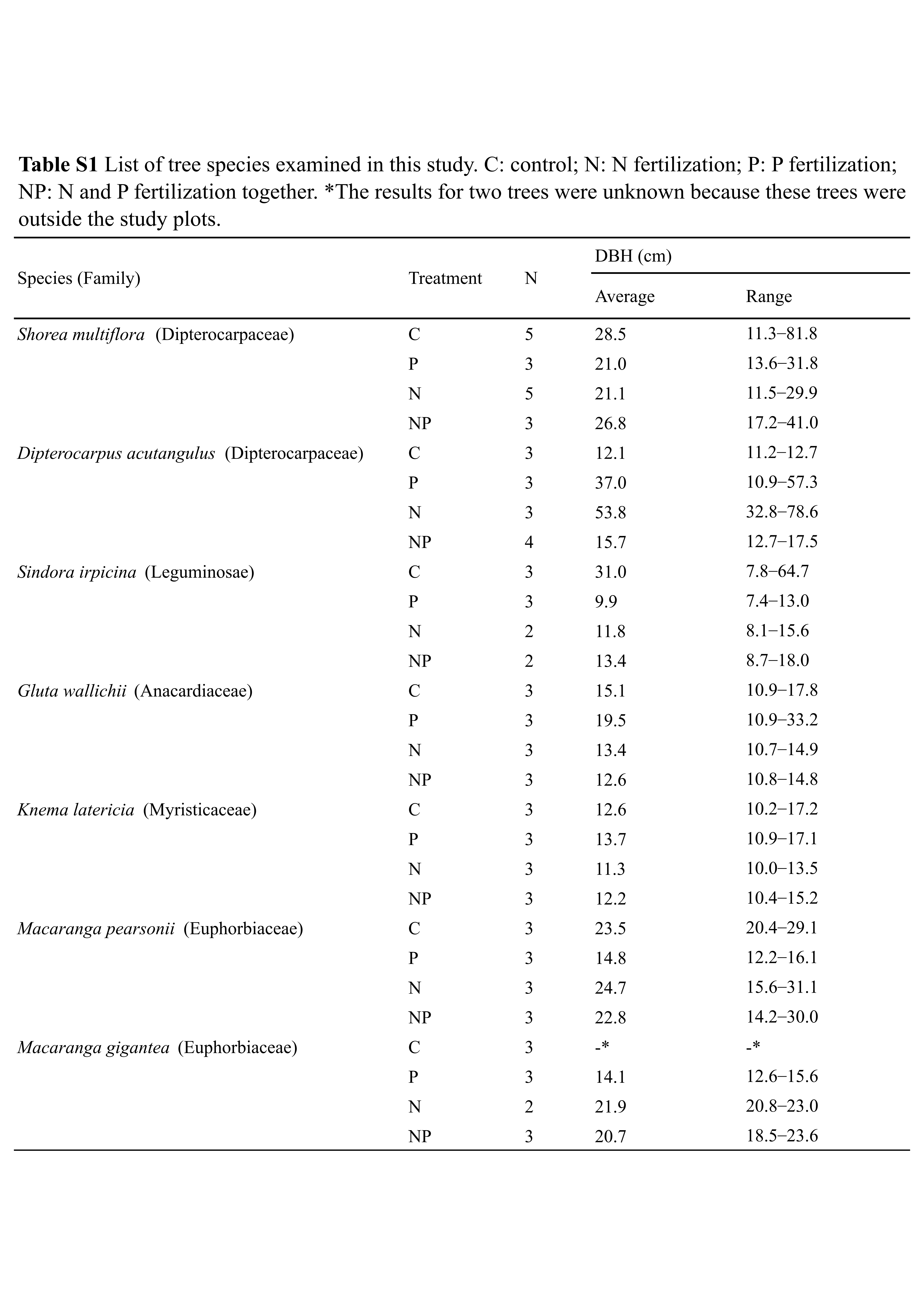

Supplement: Supplementary file 3 — Table S1 [file ECE3-12-e8669-s004.tif]

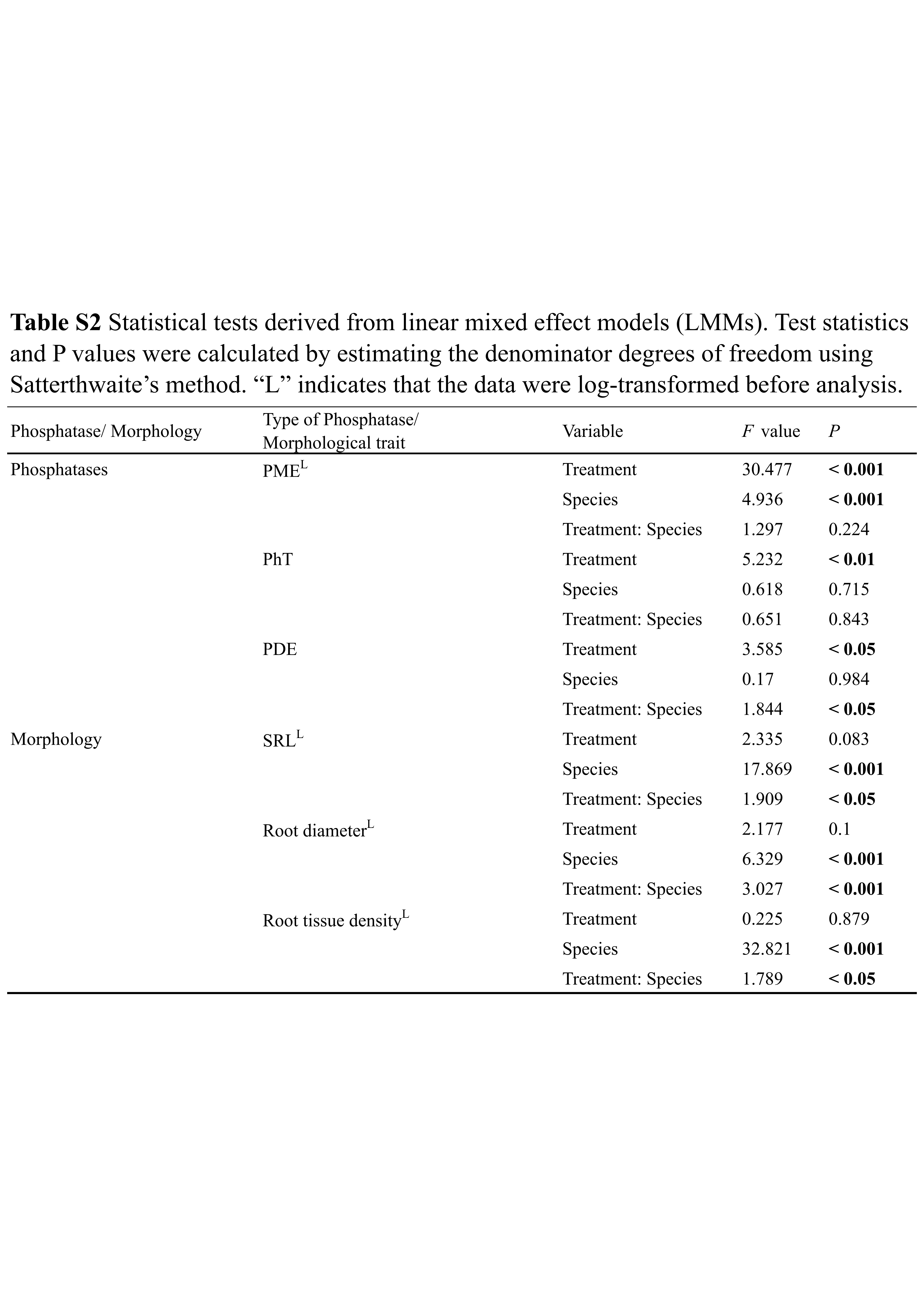

Supplement: Supplementary file 4 — Table S2 [file ECE3-12-e8669-s001.tif]
